# Antibiotic calcium sulfate-loaded hybrid transport versus traditional Ilizarov bone transport in the treatment of large tibial defects after trauma

**DOI:** 10.1186/s13018-021-02723-9

**Published:** 2021-09-20

**Authors:** Qiang Huang, Cheng Ren, Ming Li, YiBo Xu, Zhong Li, Hua Lin, Kun Zhang, Teng Ma

**Affiliations:** Department of Orthopaedic Surgery, Hong Hui Hospital, Xi’an Jiaotong University College of Medicine, Xi’an, 710054 Shaanxi China

**Keywords:** Calcium sulfate, Ilizarov, Bone transport, Tibial defects, Intramedullary nail

## Abstract

**Background:**

The purpose of this study was to compare the clinical effects of antibiotic calcium sulfate-loaded hybrid transport (ACSLHT) and traditional Ilizarov bone transport (TIBT) in the treatment of large tibial defects after trauma.

**Methods:**

Eighty-five patients with large tibial defects after trauma were selected for retrospective study. The range of tibial defects was 6–22 cm. After thorough debridement and infection controlled, bone transport technique was used to reconstruct tibial defects. Forty-four patients were treated with ACSLHT technique (the ACSLHT group), while the other 41 were treated with TIBT technique (the TIBT group). Time in external fixator was evaluated by EFI score. Enneking score was used to evaluate limb functions. SAS score was used to evaluate postoperative anxiety status. In addition, complication incidence was compared, including axis deviation, docking site nonunion, infection recurrence and so on.

**Results:**

There was no significant difference in preoperative general data between ACSLHT and TIBT group. EFI score in ACSLHT and TIBT group was 0.6 ± 0.1 cm/month and 1.7 ± 0.3 cm/month, respectively (*P* < 0.05). Enneking score of ACSLHT and TIBT group was 86.5% and 75.1% (*P* < 0.05). SAS score of ACSLHT group was significantly lower than that of TIBT group (*P* < 0.05). Complication incidence in ACSLHT group was significantly lower than that in TIBT group (*P* < 0.05).

**Conclusions:**

Compared with TIBT group, ACSLHT group had shorter time in external fixator, better limb functions, lower postoperative anxiety score and lower complication incidence which is worth of clinical promotion.

## Background

The number of patients with open tibial fracture, osteomyelitis and nonunion caused by high energy injuries is increasing year by year. In the process of surgical treatment, it is often necessary to remove all dead bones and sclerotic bones, resulting in large bone defects [1]. It is a great challenge for orthopedic surgeons to reconstruct large bone defects because of its complexity and high complication incidence [2, 3]. At present, Ilizarov bone transport technique has been widely used in the treatment of tibial segmental defects. It can effectively solve the problems of insufficient donor site, nonunion of bone grafts and limb shortening by using the principle of distraction osteogenesis [4, 5].

However, Ilizarov bone transport technique has the following limitations. Firstly, it is easy to cause axis deviation in the process of bone transport. If the two ends of bone defects are obviously not well matched, the axis may need to be adjusted by an operation. Secondly, bone transport speed is 1 mm/day, but the mineralization time is twice as long as the total transport time. This leads to a longer treatment cycle and an increased risk of poor mineralization of new bones. Thirdly, because of soft tissue cap filling, sclerotic bones and bone absorption may appear at the docking site, which leads to nonunion [6]. “Accordion technique” or an operation is needed to promote healing of the docking site if nonunion appears. Fourthly, pin infection is easy to appear in the process of bone transport. External fixator can provide infection channel for bacteria. Moreover, the long-term wearing of an external fixator is easy to cause loosening of needle tracks, leading to increased needle track infection. Therefore, patients need to take care of the needle channels regularly. Fifthly, life is inconvenient due to long-term wearing of an Ilizarov frame. In the process of bone transport, due to muscle contraction, the pressure in soft tissue increases, which can cause persistent pain and skin cutting injuries. An Ilizarov fixator is clumsy to wear and inconvenient to use. Wearing it for a long time will affect daily activities of patients and lead to anxiety of patients [7, 8]. Sixthly, patients’ compliance is also an important factor influencing the clinical effects of bone transport.

Masquelet technique is also widely used in bone defect reconstruction [9]. This technique can cure large tibial defects up to 25 cm in length, and limb functions can be recovered after an average of 8.5 months. Masquelet technique is easy to operate. It has definite curative effects, wide indications, and can improve the anti-infection ability to a certain extent. Therefore, for patients with tibial osteomyelitis and segmental bone defects, Masquelet technique has certain natural advantages. However, Masquelet technique also has some shortcomings. It usually needs surgical treatment by stages. The initial stage operation is debridement and bone cement filling, and the second stage is bone transplantation in the induction membrane. The inducer is polymethylmethacrylate (PMMA) bone cement, which needs to be removed by a secondary surgery. The heat released during the curing process of PMMA will damage the surrounding soft tissues. The elastic modulus of bone cement is significantly different from that of bones, which is easy to cause stress fractures. Moreover, for patients with large bone defects, the number of implanted autogenous bones is limited.

Calcium sulfate as a bone graft substitute has been studied for more than 100 years. A large number of experiments have proved that calcium sulfate has good biocompatibility and biodegradability [10]. Some experiments showed that osteoblasts could adhere to the wall completely and proliferate vigorously in the presence of calcium sulfate. After calcium sulfate is implanted in vivo, there are no obvious inflammation and foreign body reaction between calcium sulfate and bone interface, and there is no aggregation of neutrophils and macrophages. Calcium sulfate is absorbed by osteoclasts in vivo to form biodegradation, and the released calcium ion participates in the formation of new bones. The implantation of antibiotic-loaded calcium sulfate cement into bone defect area can effectively control infection and promote osteogenesis. We used calcium sulfate cement loaded with antibiotics to implant into the tibial defects, together with Ilizarov bone transport over an intramedullary nail or a plate, to treat patients with large tibial defects, and achieved good clinical results. The difference of the therapeutic effects between ACSLHT and TIBT group in the treatment of large tibial defects after trauma was compared in this study. It is reported as follows.

## Methods

*Inclusion criteria* (1) Patients older than 16 years; (2) patients with tibial defects after trauma; (3) patients with complete medical records; and (4) the bone defects ranged from 6 to 22 cm.

*Exclusion criteria* (1) Patients younger than 16 years; (2) patients with tibial defects less than 6 cm or more than 22 cm; (3) patients with severe medical diseases and intolerance of a surgery; (4) patients with communication disorders or history of mental illness; and (5) patients with incomplete medical records.

### General data

From January 2015 to January 2018, 85 patients with large tibial defects after trauma were selected in Xi'an Honghui hospital. There were 56 males and 29 females, aged 17–70 years. The range of tibial defects was between 6 and 22 cm. Forty-four patients were treated with ACSLHT technique, while the other 41 by TIBT technique. This study was approved by the ethics committee of Xi'an Honghui hospital. All patients or their families signed the informed consent before operation.

### Preoperative management

All patients needed to have a general examination after admission. The affected limbs were routinely examined by X-rays, and blood samples were taken to detect ESR, C-reactive protein and other infection indicators. All patients underwent one-stage debridement and repair of soft tissue defects. After infection controlled and soft tissue healing, different bone transport methods were used for bone reconstruction.

### Operation procedures of the ACSLHT group

**Fig. 1:**
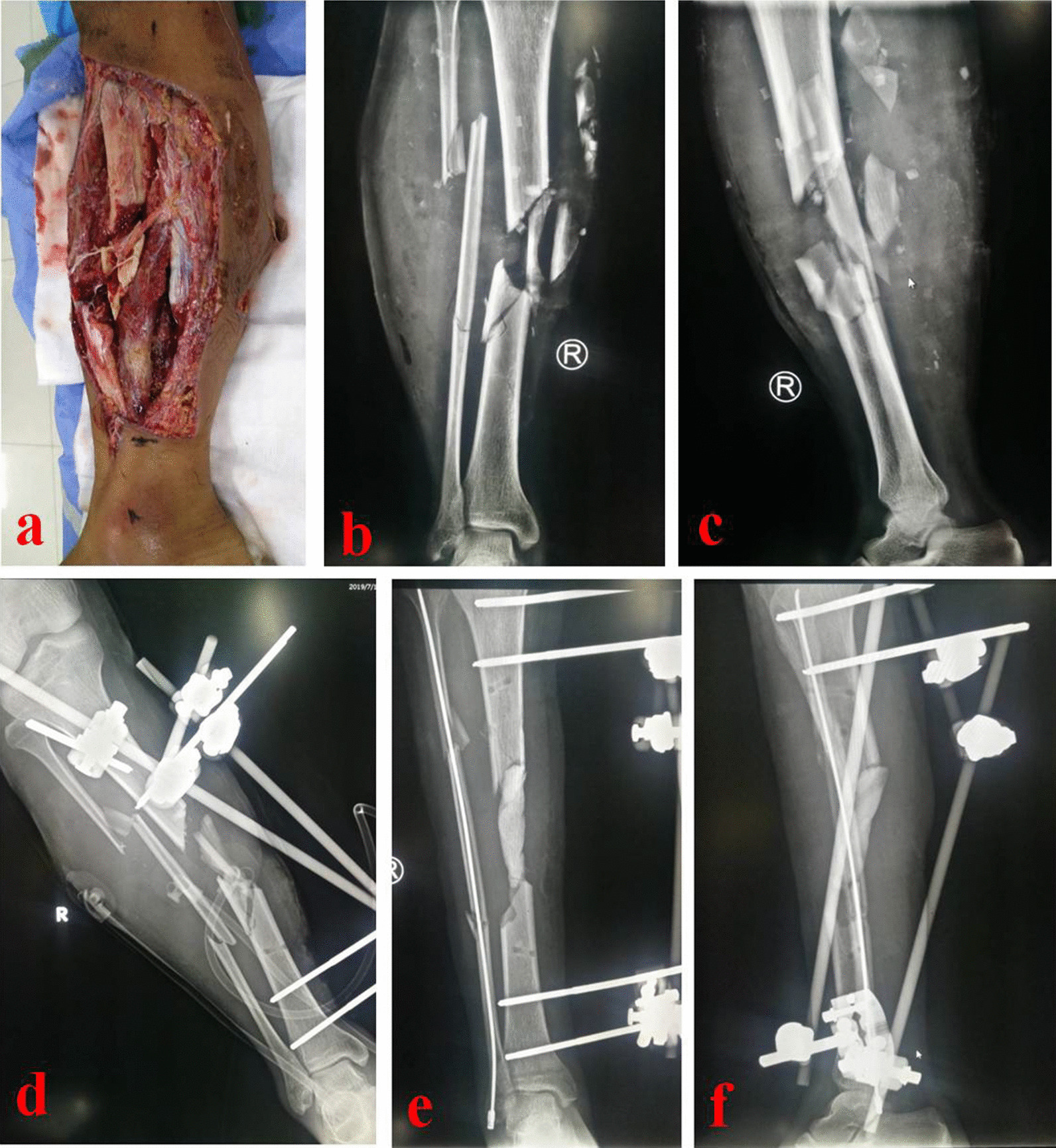
A 35-year-old male suffered from a car accident and was diagnosed as tibial open fracture. **a** Appearance before debridement, **b** and **c** X-rays of the affected tibia before debridement; **d**–**f** after radical debridement and soft tissue restoration, an 11 cm of bone defects was left

**Fig. 2:**
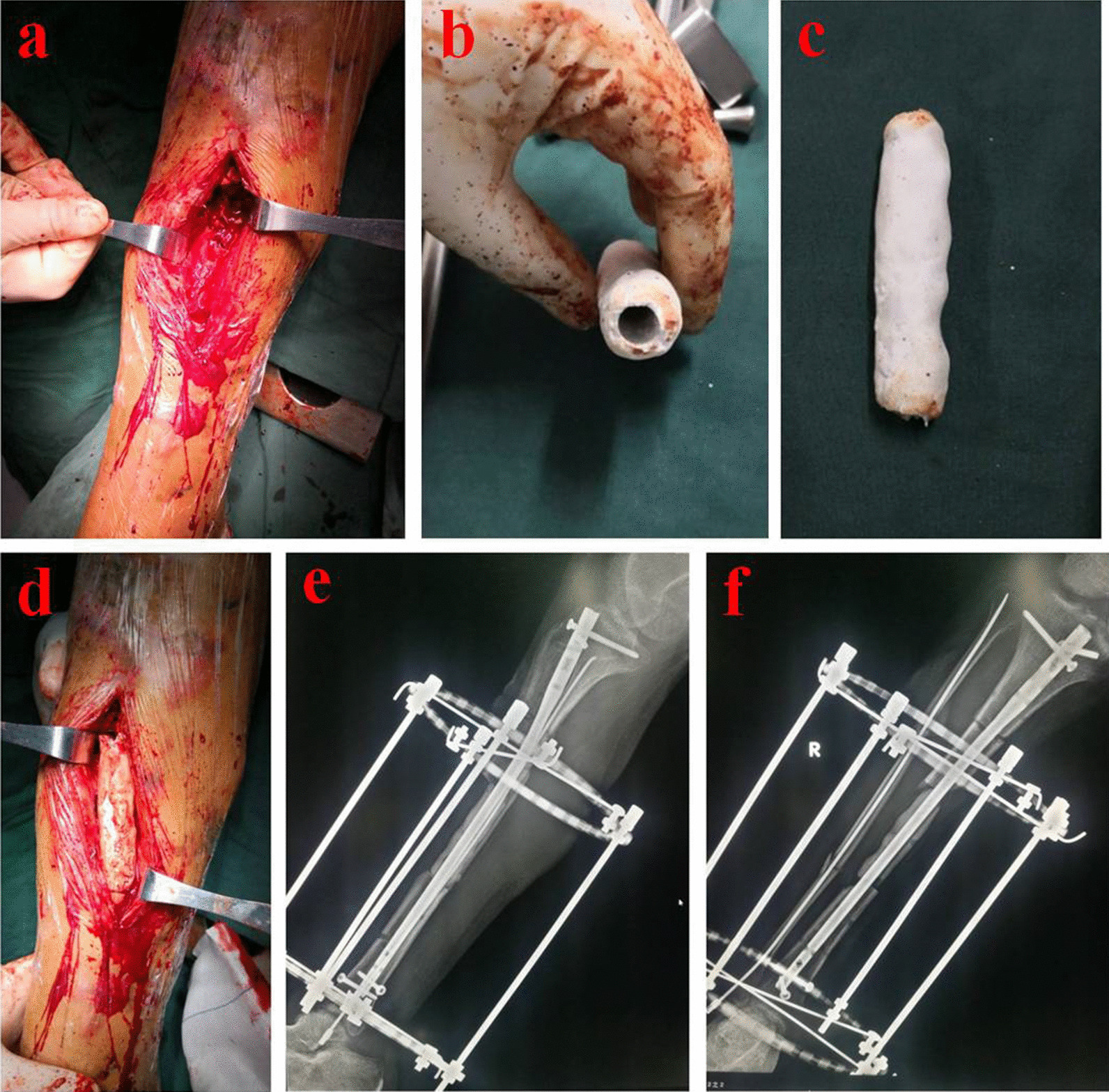
The 35-year-old patient was treated by the ACSLHT technique. **a** A large defects in the middle of tibia could be seen without infection, **b** and **c** an antibiotic-loaded calcium sulfate cement spacer was made during operation; **d** the spacer was implanted into the tibial defect area, **e** and **f** X-rays after an intramedullary nail and Ilizarov external fixator implantation. ACSLHT stands for antibiotic calcium sulfate-loaded hybrid transport

**Fig. 3:**
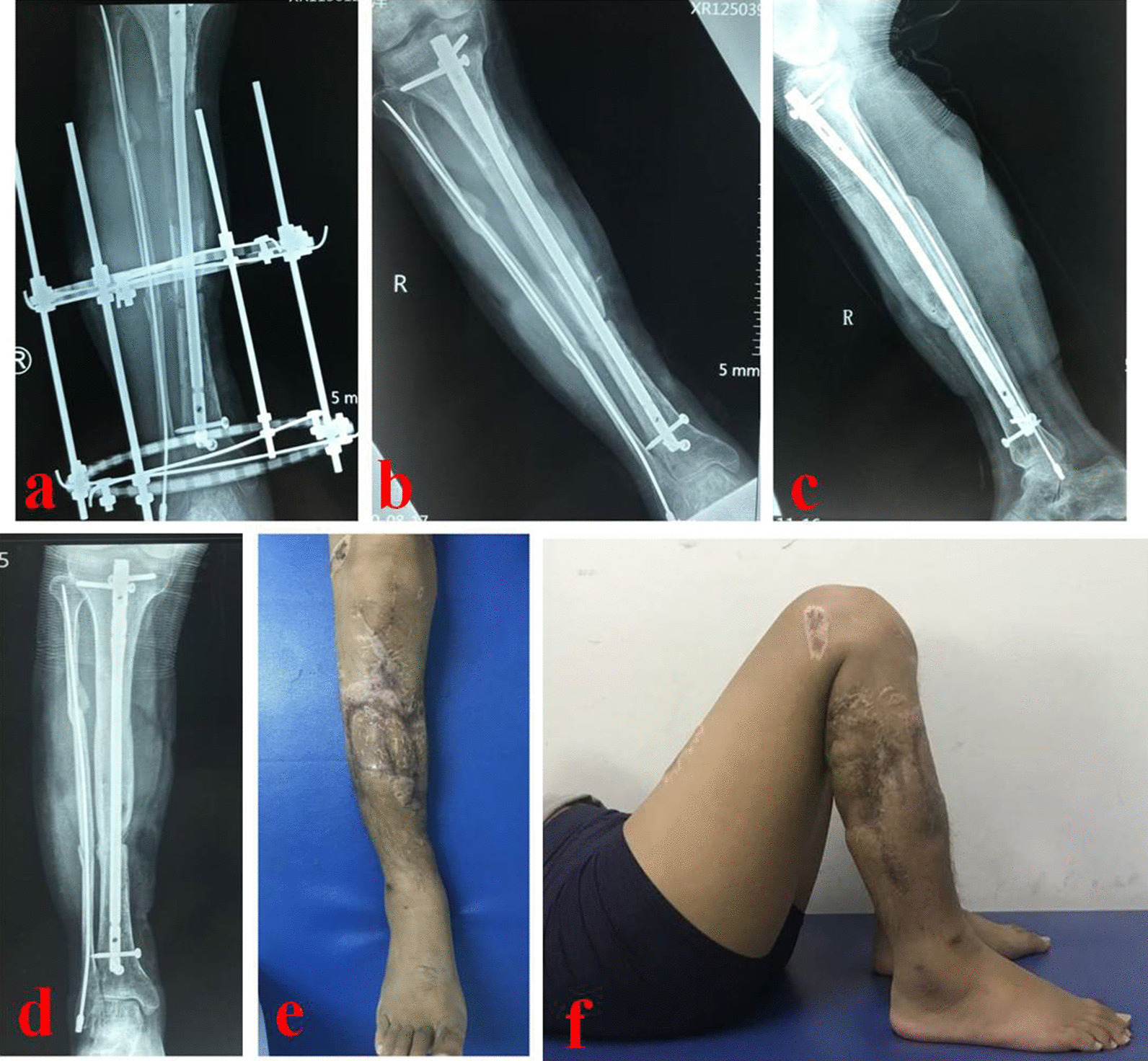
The transport process and postoperative limb functions of the 35-year-old patient. **a** The X-rays at 5 months showed that calcium sulfate cement was absorbed and new bones formed, **b** and **c** the external fixator was removed at 6 months after operation, and **d** 9 months after operation, X-rays showed that the new bone mineralization was good; e and f: postoperative appearance and knee function of the leg

**Fig. 4:**
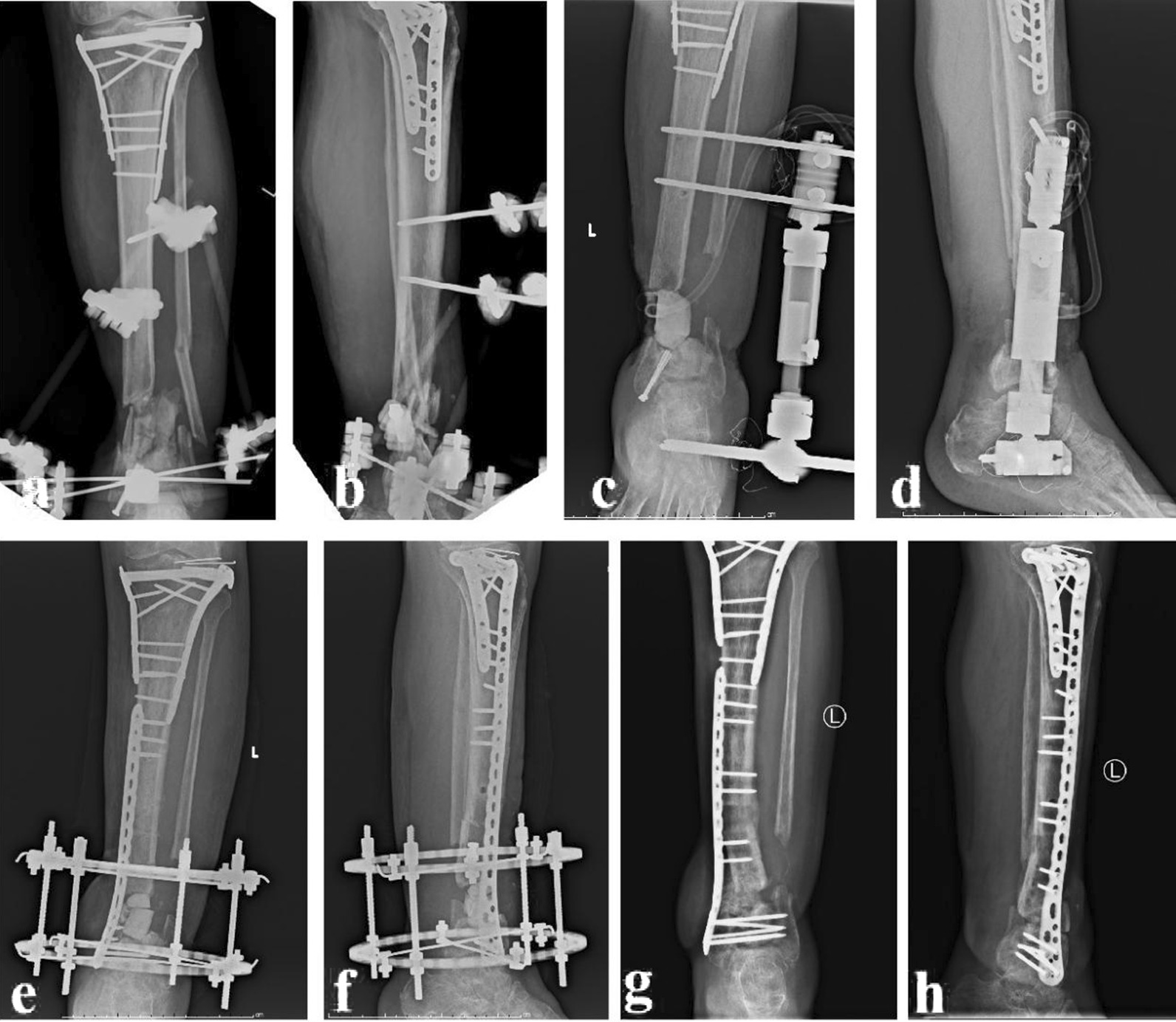
A 49-year-old male was diagnosed as osteomyelitis of distal tibia and treated with the ACSLHT technique. **a** and **b** X-rays before removal of all dead bones, **c** and **d** after debridement, 6.5 cm of distal tibial defects was observed, **e** and **f** this patient was treated with the ACSLHT technique, and X-rays showed the bone transport process, **g** and **h** X-rays after removal of the Ilizarov external fixator. ACSLHT stands for antibiotic calcium sulfate-loaded hybrid transport

A longitudinal incision was made at the anterior side of the leg with the defects as the center. The bone defect area should be exposed and repaired properly. Antibiotics and calcium sulfate were mixed in proper proportion to prepare antibiotic-loaded calcium sulfate cement spacer. Antibiotics were selected according to the results of preoperative drug sensitivity. The most commonly used ratio is: calcium sulfate 7.5 g + vancomycin 0.5 g + gentamicin injection 3 ml. The length of the spacer was slightly less than that of the bone defects by 0.5–1 cm. When an intramedullary nail was used for internal fixation, the spacer was made into a hollow pipe structure device. The diameter of the tube was slightly larger than that of the tibial intramedullary nail. When a plate was used for internal fixation, the spacer was made into a column. The length, axis and rotation of the lower limb were maintained, and the spacer was implanted into the bone defect area. According to the direction of bone transport, osteotomy was performed at the proximal or distal tibia. A tibial intramedullary nail or plate with suitable length was inserted. The intramedullary nail was inserted through the center of the bone cement spacer, and the distal and proximal ends were locked, respectively. If a plate was used, it needed to be properly fixed across both ends of the bone defect area. Then, the Ilizarov frame was installed, including 2 rings. (One was the fixation ring, and the other was the transport ring.) Several crossed Kirschner wires should be inserted to fix the Ilizarov external frame. The direction of Kirschner wires should avoid the intramedullary nail or plate. Fluoroscopy was used to confirm that the position of the intramedullary nail or plate, and the Ilizarov frame was appropriate. One week after operation, the external fixator was adjusted for bone transport. At the beginning of bone transport, the affected limb began to bear weight. After the docking site was in contact and pressed properly, the annular external fixator could be removed. Two typical cases are shown in Figs. 1, 2, 3 and 4.

### Operation procedures of the TIBT group

**Fig. 5:**
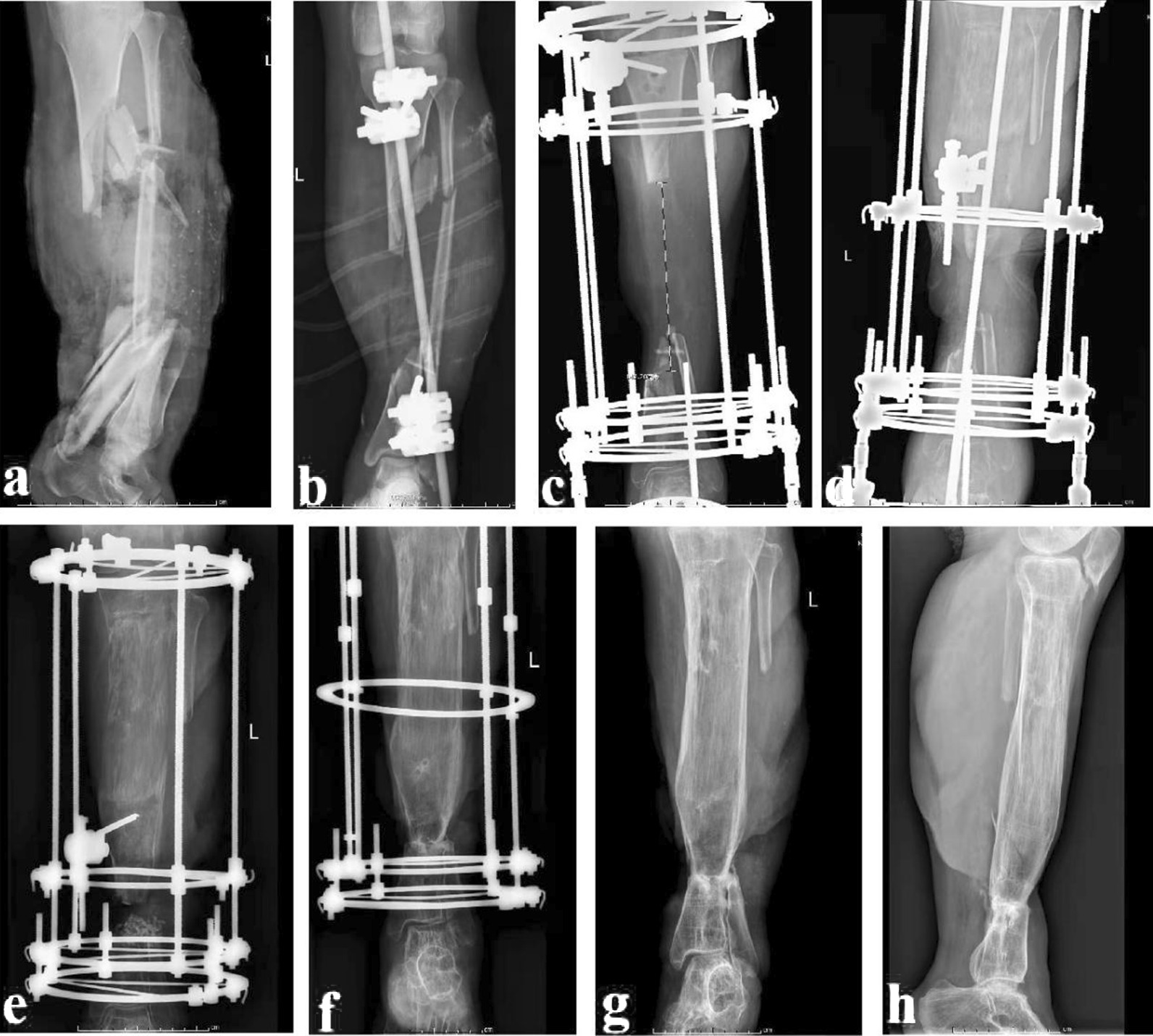
A 23-year-old female was diagnosed as severe tibial open fracture and treated with the TIBT technique. **a** X-rays after injury. **b** After debridement, X-rays of the affected tibia. **c** This patient was treated with the TIBT technique, 14 cm of tibial defects were observed. **d**–**f** The X-rays showed the bone transport process. **g** and **h** X-rays after removal of the Ilizarov external fixator. TIBT stands for traditional Ilizarov bone transport

The lower leg was maintained in the center of the Ilizarov fixator. Parallel to the knee and ankle joint surface, the Ilizarov frame was fixed with Kirschner wires. According to the surgical plan, the distal or proximal tibial osteotomy was performed. Kirschner wires were inserted to fix the moving segment. Under fluoroscopy, the bone defect alignment was adjusted to ensure that there was no axis deviation. The wound was washed and sutured. One week after operation, the external fixator was adjusted for bone transport. After the docking site was in contact, continue to press appropriately to make the docking site heal. During the process of bone transport, the condition of nerves and blood vessels should be well monitored. The Ilizarov external fixator could be removed after the docking site was firmly healed and the transport section was completely mineralized. A typical case is shown in Fig. 5.

### Postoperative management

Proper exercise of knee and ankle joint was performed after operation. Patients were treated with anti-inflammatory, detumescence, pain relief and other symptomatic treatments. All patients were treated with sensitive intravenous antibiotics for 6 weeks. According to the results of drug sensitivity, antibiotics were adjusted in time, and inflammatory indexes were monitored regularly. X-rays were rechecked every 2 weeks after operation to evaluate the effects of bone transport. All patients were followed up for at least 2 years.

### Observation indexes

The general data of the two groups were compared. The time in external fixator of the two groups was recorded. The external fixation index (EFI) was defined as time in the external fixator per centimeter of lengthening [11]. EFI score of the two groups was calculated. The limb functions of the two groups were evaluated by Enneking score [12], including limb pain, activity function, self-feeling, brace use, walking ability and gait change. Each item was scored 0–5 points with a full score of 30 points. The cumulative score divided by 30 points was the percentage of normal limb functions. The postoperative anxiety was evaluated by SAS score [13], including no anxiety, mild anxiety and severe anxiety. In addition, the postoperative complications and the number of operations needed to deal with the complications were compared between the two groups.

### Statistical analysis

SPSS 23.0 software was used to process data. Measurement data were expressed as mean ± standard deviation. Unpaired t test was used for comparisons between the two groups. Count data were analyzed using χ^2^ test. *P* < 0.05 was defined as statistically significant.

## Results

### Demographics of patients

The average age of ACSLHT and TIBT group was 39.2 ± 4.6 and 38.8 ± 4.9 years. The male patients in ACSLHT and TIBT group were 30 and 26, while female patients were 14 and 15. The range of bone defects in ACSLHT and TIBT group was 12.5 ± 3.6 cm and 13.1 ± 3.4 cm. Forty-eight patients with tibial defects were caused by acute trauma, while 37 cases by osteomyelitis. The average timing of transport after damage control surgery (DCS) was 6.2 ± 1.5 weeks and 6.1 ± 1.7 weeks in ACSLHT and TIBT group, respectively. The average times of plastic surgery were 2.4 ± 1.1 and 2.5 ± 1.3 in ACSLHT and TIBT group. Thirty-five patients had a previous open fracture in ACSLHT group; 2 were affected by a GA I, 5 patients sustained a GA II and 28 cases reported a GA III. In addition, 33 patients had a previous open fracture in TIBT group; 3 were affected by a GA I, 4 patients sustained a GA II and 26 cases reported a GA III. Fifteen patients suffered from multiple injuries in ACSLHT group, while 14 in TIBT group, respectively. There was no significant difference between the two groups in age, gender, size of bone defects, etiology, classification, etc. (*P* > 0.05, Table 1).

**Table 1:** Demographics of patients

Variable	ACSLHT group (*n* = 44)	TIBT group (*n* = 41)	*P* value
Age (year)	39.2 ± 4.6	38.8 ± 4.9	0.70
Sex			0.64
Male	30	26	
Female	14	15	
Size of defects (cm)	12.5 ± 3.6	13.1 ± 3.4	0.43
Etiology			0.95
Acute trauma	25	23	
Osteomyelitis	19	18	
Timing of transport after DCS (week)	6.2 ± 1.5	6.1 ± 1.7	0.78
Times of plastic surgery	2.4 ± 1.1	2.5 ± 1.3	0.70
Classification			
Closed fracture	9	8	0.91
GA I	2	3	0.94
GA II	5	4	0.91
GA IIIa	14	13	0.99
GA IIIb	11	12	0.66
GA IIIc	3	1	0.64
Associated injuries			1.00
None	29	27	
Multiple injury	15	14	

ACSLHT stands for antibiotic calcium sulfate-loaded hybrid transport. TIBT stands for traditional Ilizarov bone transport. DCS stands for damage control surgery. GA stands for Gustilo–Anderson

### Limb functions and mental evaluation

The time in external fixator of ACSLHT and TIBT group was 6.2 ± 1.5 month and 14.4 ± 2.6 month. The EFI score of ACSLHT and TIBT group was 0.6 ± 0.1 cm/month and 1.7 ± 0.3 cm/month. Enneking score in ACSLHT group was: 26–30 points, 24 cases; 21–25 points, 17 cases; and 16–20 points, 3 cases. The average score was 25.9, and 86.5% of normal functions was recovered in ACSLHT group. Enneking score in TIBT group was: 26–30 points, 12 cases; 21–25 points, 23 cases; 16–20 points, 5 cases; and 10–15 points, 1 case. The average score was 21.6, and 75.1% of normal functions was recovered in TIBT group. Enneking score of ACSLHT group was significantly higher than that of TIBT group (*P* < 0.05, Table 2). SAS in ACSLHT group was: no anxiety, 24 cases; mild anxiety 13 cases; and moderate anxiety, 7 cases. SAS in TIBT group was: no anxiety, 9 cases; mild anxiety, 22 cases; and moderate anxiety, 10 cases. The percentage of anxiety patients was significantly lower in ACSLHT group than that in TIBT group (*P* < 0.05, Table 2).

**Table 2:** Limb functions and mental evaluation

Variable	ACSLHT group (*n* = 44)	TIBT group (*n* = 41)	*P* value
Time in external fixator (month)	6.2 ± 1.5	14.4 ± 2.6	0.01
EFI (cm/month)	0.6 ± 0.1	1.7 ± 0.3	0.01
Enneking score	86.5% (25.9)	75.1% (21.6)	0.01
26–30	24	12	
21–25	17	23	
16–20	3	5	
11–15	0	1	
SAS score			0.01
No anxiety	24	9	
Mild anxiety	13	22	
Moderate anxiety	7	10	

EFI stands for external fixation index. SAS stands for self-rating anxiety scale

### Complications

There were 0 case of axis deviation in ACSLHT group and 10 cases in TIBT group. For patients with obvious axis deviation, the Ilizarov frame was adjusted by another surgery to correct the alignment of distal and proximal ends. In ACSLHT and TIBT group, there were 3 and 12 cases of nonunion at the docking site, respectively. “Accordion technique” was used to promote the healing of the docking site in 3 cases. The soft tissue was released and removed, and the iliac bone was grafted in 8 cases. In the other 4 patients, the docking site was repaired, the autogenous bone was grafted, the Ilizarov external fixator was removed, and the internal fixation with a plate was carried out, and finally, the docking site union was achieved. There were 0 case of infection recurrence in ACSLHT group and 3 cases in TIBT group, respectively. For patients with recurrent infection, re-debridement combined with perfusion and drainage was performed to control the infection. Finally, the infection was controlled and bone transport continued. There were 5 and 9 cases of other complications in ACSLHT and TIBT group, such as foot drop, skin depression and needle tract infection. For patients with foot drop, soft tissue release and foot drop correction were performed. For patients with skin depression, skin release surgery was performed. Moreover, for patients with needle tract infection, the fixed needle was replaced. The incidence of postoperative complications in ACSLHT group was significantly lower than that in TIBT group (*P* < 0.05, Table 3). The number of operations needed to deal with complications in ACSLHT and TIBT group was 1.3 ± 0.4 and 3.0 ± 1.2, respectively (*P* < 0.05, Table 3).

**Table 3:** Complications

Variable	ACSLHT group (*n* = 44)	TIBT group (*n* = 41)	*P* value
Complications			
Axis deviation	0(0.0%)	10(24.4%)	–
Nonunion at the docking site	3	12	0.01
Infection	0(0.0%)	3(7.3%)	–
Others	5	9	0.01
Number of operations for complications	1.3 ± 0.4	3.0 ± 1.2	0.01

## Discussion and conclusions

With the increase in high-energy injuries and the wide application of internal fixation, the number of patients with large tibial defects is increasing. Reconstruction of large tibial defects after trauma is a severe challenge for orthopedic doctors. It is still controversial how to reconstruct segmental bone defects [14]. The reconstruction technique of bone defects can be selected according to the length of defects. Bone transport technique is suitable for the reconstruction of large bone defects. The traditional bone transport technique does not use antibiotic carrier to fill the bone defects, but only uses external fixator to transport and lengthen after osteotomy. In the process of bone transport, the use of antibiotic carrier can maintain the high concentration of antibiotics, which can effectively prevent the occurrence and recurrence of infection [6]. Therefore, more and more scholars recommend the use of antibiotic carrier in the treatment of bone transport technique [6, 15].

Some scholars use antibiotic calcium sulfate-loaded bone transport to treat large bone defects and have achieved good results [15, 16]. Calcium sulfate can replace PMMA as bone defect filling material, which can be degraded and absorbed by human body without secondary removal [9]. But this technique may still lead to axis deviation in the process of transport. In addition, this method has no intramedullary nail or plate for internal fixation, so the Ilizarov external fixator cannot be removed early. In recent years, the application of “bone transport over an intramedullary nail or a plate” technique is increasing in the treatment of large bone defects [17, 18]. The internal and external fixation is carried out simultaneously which can enhance the stability of bone defect area. At the same time, it ensures that the transport section runs along the intramedullary nail or plate without axis deviation. At the end of transport, the Ilizarov external fixator can be removed, and the intramedullary nail or plate can provide stability during the initial mineralization period [19]. But internal and external fixation simultaneously will lead to disastrous consequences in case of infection. Infection is transmitted from the needle tract to the intramedullary nail or plate, leading to infection of the whole medullary cavity. This will significantly increase the number of operations, increase the cost of treatment and make patients bear more physical and mental burden. We innovatively used the antibiotic calcium sulfate-loaded hybrid transport method to treat large bone defects. Through literature review, this method has not been reported. This method combines the advantages of antibiotic calcium sulfate, intramedullary nail or plate and Ilizarov external fixator, while at the same time effectively reducing their respective disadvantages. Our data showed that, compared with the TIBT group, the ACSLHT group had shorter time in external fixator, better limb functions, lower postoperative anxiety score and lower overall complication incidence. Qin et al. found that antibiotic-loaded calcium sulfate implantation for lower chronic limb osteomyelitis was a more successful method than wound irrigation–suction, and it greatly decreased infection recurrence and docking obstruction [15, 16]. Our conclusions are consistent with this. In addition, we have properly solved the problem of axis deviation in the process of bone transport. Some scholars recommend the use of intramedullary antibiotic-coated nails in open tibial fractures to reduce the incidence of infection [20, 21]. This kind of intramedullary nail carries just a small amount of antibiotics, and the release rate of antibiotics is fast. 80% of the antibiotics are released within 24–48 h [22]. Therefore, whether this kind of coated intramedullary nail is suitable for the treatment of large bone defects remains to be verified. In our study, the antibiotic-loaded calcium sulfate can keep a high concentration of antibiotics locally for several months. These sustained-release effects are very important for the prevention of infection recurrence.

The main advantages of ACSLHT technique in the treatment of large bone defects are as follows. Firstly, this technique can avoid axis deviation in the process of transport. Because the bone transport is carried out along the direction of intramedullary nail or plate, good alignment has been maintained before the transport, so there will be no axis deviation. Secondly, this technique can reduce the occurrence of nonunion at the docking site. Calcium sulfate cement can act as a spacer in the bone defect area. It can prevent the insertion of soft tissues in the process of bone transport, thus blocking the bone transport. It is very beneficial for the transport segment to recanalize with the medullary cavity at the other end. Thirdly, this technique can reduce the incidence or recurrence of infection. Calcium sulfate can maintain a high concentration of antibiotics for a long time in local area after carrying antibiotics [23]. The release of antibiotics is accompanied by degradation of itself, which can reduce the incidence of infection. In recent years, calcium sulfate has been widely used as an antibiotic carrier in clinic, especially in the treatment of open fractures, osteomyelitis and bone defects. Some studies showed that there was no significant difference between calcium sulfate and PMMA in eliminating infection [24]. Other studies have shown that the effect of calcium sulfate carrying antibiotics was similar to or even better than that of PMMA carrying antibiotics [25]. Fourthly, this technique can significantly shorten the time of wearing an external frame. When using the TIBT method, the wearing time of the Ilizarov fixator is long, as short as a few months, as long as 2–3 years [26]. Patients feel extremely uncomfortable and have obvious anxiety and depression motion. The prolonged treatment time and extremely inconvenience have questioned its use because patient-requested amputation has been documented as a result of intolerance of the treatment [27, 28]. However, the Ilizarov fixator can be removed at the end of bone transport with the ACSLHT method, and the whole subsequent mineralization period is completed without the external fixator. This greatly shortens the time of wearing the external fixator and helps to alleviate the anxiety of patients, improve the quality of life and make patients return to normal social life and work as soon as possible.

There were still some deficiencies in this study. The number of patients in this group was small, and the follow-up time was short. Moreover, this study was a clinical retrospective study, which was still lack of large sample randomized controlled prospective study to prove it. For these deficiencies, we will discuss them in detail in further research.

## Conclusions

Compared with the TIBT technique, the ACSLHT technique had shorter time in external fixator, better limb functions, lower postoperative anxiety score and lower overall complication incidence which is worth of clinical promotion.

## Data Availability

All data analyzed in this study have been provided in the manuscript.

## Abbreviations

ACSLHT: Antibiotic calcium sulfate-loaded hybrid transport
TIBT: Traditional Ilizarov bone transport
PMMA: Polymethylmethacrylate
EFI: External fixation index
SAS: Self-rating anxiety scale
DCS: Damage control surgery
GA: Gustilo–Anderson
